# Temperature, Viral Genetics, and the Transmission of West Nile Virus by *Culex pipiens* Mosquitoes

**DOI:** 10.1371/journal.ppat.1000092

**Published:** 2008-06-27

**Authors:** A. Marm Kilpatrick, Mark A. Meola, Robin M. Moudy, Laura D. Kramer

**Affiliations:** 1 Consortium for Conservation Medicine, New York, New York, United States of America; 2 Department of Ecology and Evolutionary Biology, University of California, Santa Cruz, California, United States of America; 3 Wadsworth Center, New York State Department of Health, Slingerlands, New York, United States of America; 4 Department of Biomedical Sciences, School of Public Health, State University of New York at Albany, Albany, New York, United States of America; University of California Irvine, United States of America

## Abstract

The distribution and intensity of transmission of vector-borne pathogens can be strongly influenced by the competence of vectors. Vector competence, in turn, can be influenced by temperature and viral genetics. West Nile virus (WNV) was introduced into the United States of America in 1999 and subsequently spread throughout much of the Americas. Previously, we have shown that a novel genotype of WNV, WN02, first detected in 2001, spread across the US and was more efficient than the introduced genotype, NY99, at infecting, disseminating, and being transmitted by *Culex* mosquitoes. In the current study, we determined the relationship between temperature and time since feeding on the probability of transmitting each genotype of WNV. We found that the advantage of the WN02 genotype increases with the product of time and temperature. Thus, warmer temperatures would have facilitated the invasion of the WN02 genotype. In addition, we found that transmission of WNV accelerated sharply with increasing temperature, T, (best fit by a function of T^4^) showing that traditional degree-day models underestimate the impact of temperature on WNV transmission. This laboratory study suggests that both viral evolution and temperature help shape the distribution and intensity of transmission of WNV, and provides a model for predicting the impact of temperature and global warming on WNV transmission.

## Author Summary

West Nile virus (WNV) was introduced into New York in 1999 and subsequently expanded its range to include much of North, Central, and South America. Previously, we have shown that a new strain of WNV (referred to as WN02) that was first detected in 2001 and subsequently spread across North America was more efficient at infecting and being transmitted by *Culex* mosquitoes than the strain that was originally introduced (referred to as NY99). In the current study, we determined how temperature and time since feeding on infected blood affected the probability that mosquitoes would transmit these two strains of WNV. We found that the advantage of the WN02 strain over the NY99 strain increased with both temperature and time. Thus, warmer temperatures would have facilitated the invasion of the WN02 strain. In addition, we found that transmission of both strains of WNV accelerated sharply with increasing temperature, such that small increases in temperature had relatively large effects on transmission. This laboratory study suggests that both viral evolution and temperature influence the distribution and intensity of transmission of WNV, and provides a model for predicting the impact of temperature and global warming on virus transmission.

## Introduction

The interaction between pathogens, their vectors, and vertebrate hosts is a dynamic one, and evolution in any one of the three can significantly alter transmission dynamics. Theory suggests that pathogens will evolve to maximize their fitness, which is a function of transmissibility and virulence to the host [1],[2]. Pathogens that infect and replicate well in their vertebrate hosts and vectors may decrease the survival of both which may reduce their lifespan for transmission.

At the same time, the distribution and intensity of transmission of vector-borne pathogens is strongly influenced by the interaction of temperature, vectors, hosts, and pathogen genetics. Temperature can determine both the latitudinal boundary and upper elevational limit of pathogen transmission if the extrinsic incubation period (EIP) is greater than the longevity of the vector [3]. Temperature also has been linked to changes in the intensity of transmission of pathogens [4],[5], which may be linked to temperature-induced changes in the EIP, the longevity, and the feeding rate of vectors [6],[7].

West Nile virus (WNV; *Flaviviridae, Flavivirus*), is a single-stranded positive-sense RNA virus that was introduced into the western hemisphere in 1999 and has subsequently spread throughout much of North, Central, and South America [8],[9]. It is primarily transmitted between birds (especially American robins, *Turdus migratorius*, in many areas [10]–[14]) and *Culex* mosquitoes [8],[15] and has caused at least 2,500 reported cases each year since 2002 for a total of 32,135 total reported cases, 11,243 cases of encephalitis, and 1,125 deaths, with an estimated 1.56 million infections and 310,000 illnesses from 1999–2007 [9], [16]–[18]. In addition, WNV has evolved over the past 7 years, and a genotype that was first isolated in 2001 (termed WN02) has displaced the introduced genotype (termed NY99) [19],[20]. WNV strains in the WN02 or North American dominant genotype have three consensus changes in the full length genome compared to NY99 [19],[20]. The rapid expansion of the WN02 genotype has been linked to a shorter extrinsic incubation period in *Culex* mosquitoes [21],[22], but the full mechanisms of displacement are not yet known. In particular, in previous studies WN02 genotypes were transmitted more efficiently than NY99 by *Cx pipiens* on 5 and 7 days post feeding, but not day 9 and by *Cx. tarsalis* from 5 to 14 days post feeding [21],[22].

The vector competence of mosquitoes characterizes their ability to transmit a pathogen after taking an infected blood meal. The fraction of vectors transmitting the pathogen is known to vary between populations of a species [23]–[25], and increase with time [26],[27] and temperature for WNV [28]–[30] and many other pathogens, including western equine encephalomyelitis virus and St. Louis encephalitis virus in *Cx. tarsalis* [28],[31], Rift Valley Fever virus in *Aedes fowleri* [32], Ockelbo virus in *Culex* spp [33] and *Aedes* spp [34], and African horse sickness virus, bluetongue virus, and epizootic hemorrhagic disease in *Culicoides sonorensis* [35]. However, the exact relationship between vector competence, temperature and the time since feeding on an infected host is not clear, and in other studies the influence of temperature on vector competence varies, sometimes depending on the mosquito species infected [33],[36],[37].

A degree day model developed for *Cx. tarsalis* has been used to model the effect of temperature on WNV transmission across North America [28],[38]. In this approach, a mosquito (or a fraction of a population of mosquitoes) feeding on an infected host becomes infectious after a time period at a certain temperature, termed the number of degree days. Degree days are often measured as the number of days since feeding multiplied by the temperature in degrees Celsius above a minimum temperature threshold (*T_thr_*) below which no transmission is assumed to occur. However, the exponential increase with temperature in chemical and molecular processes contributing to viral replication would suggest that the relationship between transmission and the number of degree days should be accelerating and would not be well described by a simple degree day model. Transmission would be expected to be higher at higher temperatures given the same number of degree days. For example, if *T_thr_* = 14°C, transmission would be expected to be higher after seven days at 30°C (16° above the threshold temperature of zero transmission) than after 16 days at 21°C (7° above the *T_thr_*), even though the same number of degree days, 112, is the same in both cases. Here we explore the relationship between temperature and transmission of two genotypes of WNV (NY99 and WN02) and test the adequacy of a simple degree day model for WNV transmission by *Culex pipiens*, a key enzootic and bridge vector for WNV in the northern USA [15],[39].

## Methods

### Viruses and mosquitoes

Two strains of WNV were used, one belonging to genotype NY99 and one to WN02 (strain designations are NY99-3356 and WN02-1956, Genbank accession number, AF404756 and AY590210, respectively). Previous work suggested that there was little phenotypic variation between strains within genotype [21],[22]. NY99-3356 was passed twice in Vero (African Green Monkey kidney) cells, and WN02-1956 was passed once in Vero cells followed by one passage in C6/36 (*Aedes albopictus*) cells prior to use in these studies.

Colonized *Cx. pipiens* were reared and maintained in the Wadsworth Center Arbovirus Laboratory BSL-2 insectary. The colony was established in 2002 from egg rafts collected in Pennsylvania (courtesy of Michael Hutchinson) and has been maintained continuously using defibrinated goose blood (Hema Resourse and Supply, OR) for egg production and 10% sucrose ad lib for maintenance at 27°C with 16∶8 L∶D light cycle and 85% humidity. All experiments with infectious virus were performed in the BSL-3 laboratories or insectaries at the Wadsworth Center Arbovirus Laboratories.

### Vector competence

Seven day-old mosquitoes were deprived of sucrose and water for 48 h and then fed on a suspension of defibrinated goose blood (Hema Resourse and Supply, OR) plus a final concentration of 2.5% sucrose and either a NY99 or a WN02 virus, using a Hemotek feeding system (Discovery Workshops, UK). The WNV titer in the bloodmeals was 1.2–1.4×10^8^ plaque-forming units (PFU)/ml. Mosquitoes were allowed to feed for up to 1.5 h at which time engorged mosquitoes were separated from unfed mosquitoes under CO_2_ anesthesia. Fully engorged mosquitoes were placed into 0.6L cardboard cartons, supplied with 10% sucrose ad lib, and held at the prescribed temperatures under 85% RH, photoperiod of 16∶8 (L∶D). Groups of 25 mosquitoes were removed at several different intervals post-feeding and anesthetized with triethylamine (Sigma, St. Louis, MO). The days sampled included days 4, 7, 10, 14, 18, 21, 24, 28, 31, 34, and 40 for 15°C, 18°C, and 22°C and additional early sampling at days 0.5, 1, 1.5, 2, 2.5, 3 for experiments at 32°C. Legs were removed and placed in 1.0 ml of mosquito diluent (MD; 20% heat-inactivated fetal bovine serum [FBS] in Dulbecco's phosphate-buffered saline plus 50 *u*g/ml penicillin/streptomycin, 50 *u*g/ml gentamicin, and 2.5 *u*g/ml Fungizone) and frozen at −80°C for subsequent assay. Salivary secretions were collected using a modified *in vitro* capillary transmission assay [40]. Briefly, mosquito mouthparts were inserted into a capillary tube containing approximately 10 µL of a mixture of 50% sucrose and FBS (1∶1) for 30 minutes, at which time the contents were placed into 0.3 ml MD in a microfuge tube. Bodies were placed in 1.0 ml MD and all samples were frozen at −80°C for subsequent assay. Bodies and legs were homogenized separately using a mixer mill (Qiagen, Valencia, CA) at 24 cycles/s for 30 s and then clarified by centrifugation. Samples were analyzed for the presence of infectious virus by plaque assay on Vero cells as previously described [41].

### Statistical analyses

We treated each group of 25 individual mosquitoes tested after a fixed time at a temperature as an experimental unit (data point) and the fraction of mosquitoes that were infected (# with virus in the body/# fed), had disseminated infections (# with virus in their legs/# fed), or transmitted (# expectorating virus/# fed) as dependent variables. We built regression models (using SPSS v 15.0) including degree days (DD = tT, where t = time or days since feeding, and T = temperature in degrees Celsius) and a genotype by DD interaction (to test for a temperature and time varying advantage of WN02) as independent variables. We note that this statistical model (and the one described below) assumes that infection and transmission are increasing functions of temperature and time since feeding, and statistical effects model differences between genotypes as differences in the rates of increase (slopes), rather than fixed differences (intercept or main effects). We believe intercept differences are less biologically realistic because infection, dissemination, and transmission all start at zero and increase with time and temperature. In essence, the statistical effect of viral genotype is assumed to influence the rate at which the probability of a group of mosquitoes transmitting and becoming infected increases with temperature and time. We also note that our degree model implicitly assumes the minimum temperature threshold is 0°C (since it uses the raw temperature), which is likely too low, as no transmission was observed at 10°C in *Cx. tarsalis* held for 110 days [28]. However, the fit of the data were much better using raw temperature than either (Temperature −10°C) or (Temperature −14.3°C) (the residual error from models in Table 2 with a threshold of 14.3°C and 10°C were 3.02 and 1.87, respectively, compared to 0.99 with a threshold of 0°C; all regressions had the same number of predictors). We arc-sin square-root transformed the three dependent variables to normalize the residuals. We omitted an intercept from the model because we assumed that, except for residual virus in the blood meal, infection and transmission would be 0 at degree-day 0. The qualitative conclusions presented below were identical using an intercept.

We then tested the hypothesis that infection, disseminated infection, and transmission should accelerate with increasing temperature faster than the DD model, by regressing the residuals of the previous models against temperature. *A priori*, we hypothesized that the increase in transmission and infection with temperature would be a balance between chemical and kinetic processes that increase exponentially (i.e. as *e^T^* where *T* is temperature, and *e* is the base of the natural logarithm) and rate-limiting processes that would constrain viral replication. Since the residuals were significantly correlated with temperature for all three dependent variables (see Results) we attempted to determine if DD with a higher order temperature term would provide a better fit to the data. To facilitate model comparison we replaced the DD model term (*tT*) with a term that was the product of the days since feeding (t) and temperature (T), raised to the power *n* (*tT^n^*), and compared models with increasing *n*. For the DD by genotype interaction we also used DD with temperature also raised to the *n^th^* power. Finally, the qualitative results of both our analyses were unaffected by using actual temperature to calculate degree days, or degrees above a previously reported [28] threshold for zero transmission of 14.3°C. Our own data, and those in ref. [28], show that low-level transmission occurs at 14–15°C (but after very long periods that may exceed mosquito lifespan in the field).

## Results

**Figure 1:**
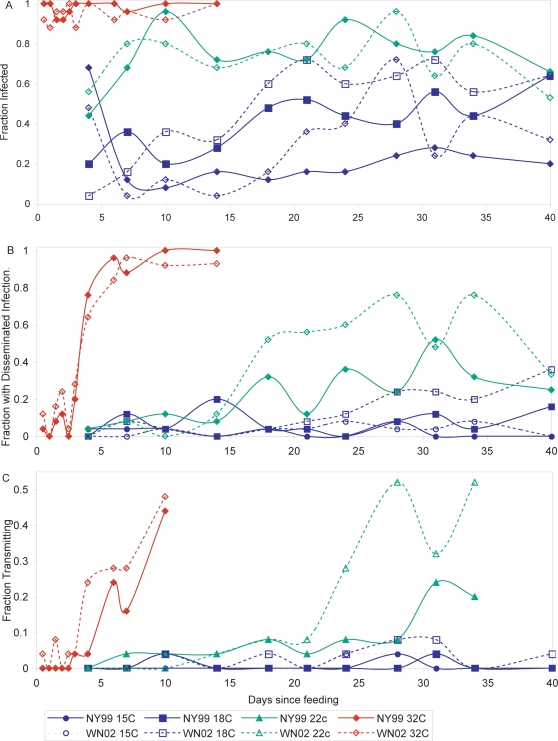
The relationship between genotype (NY99 and WN02), temperature, and days since feeding and the fraction of *Culex pipiens* mosquitoes infected (A), with disseminated infections (B), or transmitting WNV (C), after 0.5–40 days as the proportion of mosquitoes tested.

We examined a total of 2075 *Cx. pipiens* mosquitoes in 83 groups of 25 individuals and examined midgut infection, disseminated infection, and transmission from 12 hours to 40 days post-feeding (Figure 1). At 32°C, we detected transmission at 12, 36, and 60 hours for the WN02 genotype, and on day 3 (72 hours) for the NY99 genotype of WNV (Figure 1C). Virus was also present in the legs (and abdomens) of these mosquitoes at these time points (Figure 1B) and was not present in the saliva of any of the mosquitoes that were not infected, so it is unlikely that mechanical transmission or regurgitation accounted for the virus detected in the transmission assays (see also Discussion, below).

**Figure 2:**
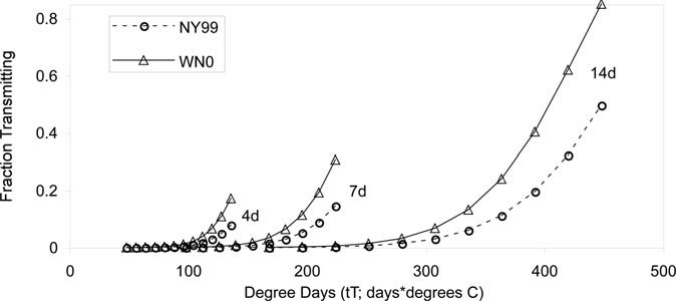
Fitted relationships between the fraction of mosquitoes transmitting virus for two genotypes of WNV and time and temperature, based on the statistical model in Table 2(WN02: Tr = (sin(8.00tT^4^/10^8^))^2^; NY99: Tr = (sin(5.32tT^4^/10^8^))^2^. Each curve shows the fraction of mosquitoes transmitting at a fixed time period after feeding on WNV-infected blood (4, 7 or 14 days) with points showing increasing temperatures (12°C to 32°C, symbol every 2°C).

The initial regressions indicated that the fraction of mosquitoes infected and the fraction with disseminated infections increased with degree days (DD = tT) since feeding (Figures 1,2; Table 1). However, neither was significantly different between genotypes (Figures 1,2; Table 1). In contrast, transmission of WNV by *Cx. pipiens* mosquitoes was significantly influenced by both DD and a genotype by DD interaction (Figures 1,2; Table 1). The coefficient of this last term indicated that the fraction of mosquitoes transmitting WNV increased faster for the WN02 genotype than the NY99 genotype (and the fitted function for WN02 was greater at all times and temperatures since both lines intersect the origin). In fact, the fraction of mosquitoes transmitting the WN02 genotype was greater than or equal to the fraction transmitting the NY99 genotype for all but two of the 42 time-temperature samplings (the two exceptions were at 22°C on days 7 and 10 where 1/25 mosquitoes transmitted the NY99 genotype but 0/25 transmitted the WN02 genotype). Thus, the WN02 genotype appeared to have an advantage at both high and low temperatures, and this advantage increased with time and temperature.

**Table 1:** Regression analysis (no intercept) of midgut infection, disseminated infection, and transmission after arc-sin square root transformation with Degree Days (DD) and a genotype (GT) by Degree Day interaction as predictors.

Term	Transmission	p-value	Disseminated Infection	p-value	Infection	p-value
DD*10^3^	0.62±0.084	<0.0005	1.20±0.16	<0.0005	2.0±0.28	<0.0005
GT-DD*10^3^	−0.27±0.12	0.029	−0.32±0.23	0.17	−0.11±0.39	0.77
Residual Error	3.41		12.9		37.5	
Total Error	6.38		24.7		82.9	

Coefficient±1SD is given.

However, the residuals of regressions for all three dependent variables was significantly correlated with temperature (all p<0.001), suggesting that a degree day predictor using a linear product of temperature and incubation period (*tT*) was not fully capturing the temperature-dependent acceleration in infection and transmission. In the second statistical analysis, we found that both transmission and disseminated infection was best predicted by a model including DD with temperature raised to the 4^th^ power (tT^4^), and a DD by genotype interaction (Table 2). The results were the same if the fraction transmitting was expressed as the fraction of *infected* mosquitoes transmitting (arc-sin square root transformed fraction of infected transmitting, DD: 9.09×10^−8^ tT^4^; p <0.0005; DD-genotype interaction: −3.30×10^−8^; p<0.0005). Infection was also best predicted by a model including DD with temperature raised to the 4^th^ power (tT^4^), but was not significantly influenced by the DD by genotype interaction (Table 2). The residual error in these second set of regressions was substantially lower compared to the first statistical analysis, with the same number of independent variables (Tables 1,2). The significant negative coefficient for DD by genotype interaction term for transmission and disseminated infection again indicates that the fraction of *Cx. pipiens* infected with and transmitting genotype WN02 increased faster than mosquitoes transmitting NY99, as illustrated by the raw data (Figure 1), and the fitted relationships (Figure 2). Thus, WN02 would have a significant advantage over NY99 under warmer conditions after the same incubation period.

**Table 2:** Regression analysis (no intercept) of midgut infection, disseminated infection, and transmission after arc-sin square root transformation with Degree Days (DD) and a genotype (GT) by Degree Day interaction as predictors, as in Table 1, except DD term was *tT^4^* (t = days since feeding on WNV-infected blood; T = temperature).

Term	Transmission (*tT^4^*)	p-value	Disseminated Infection (*tT^4^*)	p-value	Infection (*tT^4^*)	p-value
DD*10^8^	8.00±0.46	<0.0005	14.7±0.80	<0.0005	22.2±2.3	<0.0005
GT-DD*10^8^	−2.68±0.65	<0.0005	−2.2±1.1	0.05	0.43±3.2	0.89
Residual Error	0.99		3.0		24.2	
Total Error	6.38		24.7		82.9	

Coefficient±1SD is given.

## Discussion

The relationship between temperature and the transmission of pathogens has gained substantial attention recently, because projected changes in global temperature may increase the health burden of some diseases [42]. We have shown that, in the laboratory, increases in temperature have a two-fold impact on WNV transmission. First, as has been shown previously, increasing temperatures significantly increased viral infection, dissemination, and transmission, most likely through increased viral replication. Our study used the plaque assay which measured the presence of infectious virus and not the presence of unpackaged viral RNA, to test for infection, dissemination, and transmission in the mosquito. As a result, since the replication cycle is completed more quickly at higher temperatures, this will lead to greater concentration of infectious virus above the limit of detection in each mosquito. This is the case for replication in all tissues, and as such, increased temperature would affect not only infection kinetics, but dissemination and transmission kinetics as well.

Second, warmer temperatures increased the advantage of the WN02 genotype over the NY99 genotype virus, and this advantage accelerated with temperature. Thus, the WN02 genotype appears to be better adapted to warmer temperatures than NY99, and NY99 was better adapted to warm conditions than a South African strain of WNV in *Cx. tarsalis* [28]. This result highlights the importance of understanding vector-pathogen-environment interactions and the role of pathogen evolution in influencing transmission.

We also have shown that the advantage of WN02 over the NY99 genotype extends beyond day 7 post infection in *Cx. pipiens*, as we had observed in *Cx. tarsalis* [22]. The disparate results between our study and previous research that indicated no difference on day 9 [21] is likely due to extending the experiments past day 9 (up to day 40 at some temperatures) and including additional experiments resulting in much larger sample sizes. Nonetheless, our results support the earlier assertion that the WN02 genotype has an advantage over the NY99 genotype in the laboratory.

Our results refine the WNV temperature-transmission relationship and show that WNV transmission in mosquitoes accelerates nonlinearly with the extrinsic incubation temperature, suggesting that even a small increase in temperatures can have a significant impact. They show that traditional degree day models for WNV may not accurately describe the impact of temperature on transmission. Instead, transmission may be more accurately modeled using degree day functions that include a temperature term raised to a power greater than 1. For WNV, we found that a degree day term with temperature raised to the fourth power, *tT^4^*, was most accurate in explaining variation in transmission in our data. The implications of this difference are that even relatively small changes in temperature (e.g. the 2°C projected change in global temperatures [43]) have the potential to substantially increase transmission, and traditional degree day models used to investigate the potential impact of global warming will thus underestimate the effects of warming on transmission of WNV by mosquitoes. For example, if we fit a linear degree day model, *tT*, to our data, an increase from 28°C to 30°C would be predicted to increase temperature only 0.9% (from 12.1% to 13.0%), whereas the fitted model with *tT^4^* this increase from 28°C to 30°C would actually increase transmission 7.8% (from 11.4% to 19.2%).

In our study, we occasionally detected infectious virus in both salivary secretions and the legs of mosquitoes only 12 hours after feeding on an infected blood meal. Although under normal conditions the WNV replication cycle requires 10–12 hours [44], it is known that the virus replicates more quickly at higher temperatures [45]. Thus it may be possible that sufficient levels of replication took place in some mosquitoes held at 32°C to result in dissemination and transmission very quickly after feeding. It is equally possible that at high temperatures the cell junctions of the epithelium of the midgut were disrupted or increasingly permeable creating a rapid mechanism for midgut escape, possibly via leakage of virus, as has been observed with other virus-mosquito pairs [46]–[48]. This would have facilitated early escape of the virus to the legs, and subsequent infection of the salivary glands. Since the only mosquitoes that had virus in their salivary secretions were those that had virus in their legs, this argues against either mechanical transmission due to residual virus on the proboscis or regurgitation of virus from the midgut during the capillary transmission assay. Nonetheless, we cannot entirely rule out these other explanations, and furthermore, it is unlikely that mosquitoes would feed again 12 or 24 hours after the initial blood meal unless they had only obtained a partial or interrupted blood meal.

It should be noted that our study did not evaluate the impact of mosquito rearing temperature, as all immature stages were maintained at 27°C. Previous work showed that vector competence for several flaviviruses, including Murray Valley [49], Japanese encephalitis [50], and St. Louis [51] encephalitis viruses, and dengue [52] and yellow fever [53] viruses, was depressed by maintaining adults at temperatures lower than those they experienced during larval development. In contrast, transmission of two alphaviruses, eastern equine encephalitis [54] and western equine encephalomyelitis [55], were not observed to decrease when adults were maintained at temperatures lower than the rearing temperature, and early season populations were considerably more susceptible to infection that those collected during midsummer.

These results contribute to our broader understanding of how factors can generate spatial and temporal variation in transmission of pathogens. The transmission of WNV by *Cx. pipiens* has been shown to be influenced by host availability [12], mosquito genetic ancestry [56], and now the interaction of temperature and viral genotype. A key goal of future research will be to link the temperature-transmission patterns observed in the laboratory to patterns of transmission in the field. This should enable more accurate predictions of the impact of climate and climate change on the transmission of WNV and other vector borne pathogens.
